# The Story of the Hospitals

**Published:** 1887-02-12

**Authors:** 


### The Story of the Hospitals.

(*Continued from p.* 297.)

### The Cancer Hospital, Brompton.

Cancer Hospitals.

There is no malady more fearful in character, more stubborn to treatment, more terrible in the suffering it causes than cancer; yet, strange to say, the provision which the metropolis provides for its treatment is of the most limited description. Besides the hospital of which I am about to speak, together with the special wards at the Middlesex Hospital, there has really been no attempt made to grapple with this disease in a manner at all adequate, despite the fact that the resources of existing institutions for the treatment of this disease are being taxed more and more.

By-gone Brompton.

A recent visit which I paid to the Cancer Hospital at Brompton, though it brought me face to face with this disease in all its terrible reality, afforded me so much interest and profit that I cannot forbear a brief narration of the history and work of this institution. Brompton, which is still as famous for its genial air as it was in the days when the great Lord Burleigh resided here, is no longer an isolated hamlet, the chief habitat of nurserymen and market-gardeners. The rapacious builder—for he is still much as Horace has described him to us—has run to earth the cultivator of the ground, and erected thereon his "spacious," "eligible," and "desirable" residences. "Letters from Brompton," writes Addison, in No. 452 of the *Spectator*, "advise us that the Widow Blight had received several visits from John Milldew, which affords matter of speculation in those parts." How cleverly, in these few lines, does the great essayist indicate the dull monotony in which the village folk of Brompton lived in 1712. The main roads in those "good" old days were very sloughs of despond, while cart-wheels sank deeply in the ruts. How often did the cumbrous carriage of the Duchess of Munster get stuck in the mud of the Fulham Road, as she journeyed to Kensington or St. James's to pay her court to that ill-cultured monarch, King George I! But I must leave the lovely Melusina to extricate herself from the mud-holes of the Fulham Road, and hasten on.

Story of the Founding.

The Cancer Hospital at Brompton was called into existence through a circumstance of a painful character. Mrs. William Marsden, the wife of the future founder, was, about the year 1840, attacked by this terrible malady. Her husband called in the highest medical talent which England then afforded, but he found that the faculty could do absolutely nothing. Such cases as this were not then readily taken in at the general hospitals, and the study of the disease was altogether in its infancy. In the end Mrs. Marsden died, after much suffering, borne with calm, almost stoical, resignation. Dr. Marsden, though he had lost his dearest friend, determined to see what he could do to remove the stigma of ignorance which then rested on the medical profession in reference to this insidious scourge. His first step was to hold a preliminary meeting at his house in Lincoln's Inn Fields. "Now, gentlemen," he said, "I want to found a hospital for the treatment of cancer, and for the study of the disease, for at the present time we know absolutely nothing about it." A committee was formed, of whom but one man now remains, viz., Robert Francis Cooke, and a start, in a very small way, was made. This was in 1851.

Early Days of the "Cancer."

The first quarters of the Cancer Hospital comprised a modest tenement in Canon Row—old Channel Row—Westminster. The young institution soon attracted the attention of the benevolent. Here the first cancer hospital in London was housed for a couple of years, but Dr. Marsden soon found he was "cabin'd, cribb'd, confin'd." New premises were accordingly sought, and a large house at the corner of Hollywood Road, Brompton, possessing an acre of garden at the rear, was taken. There the hospital was carried on for a considerable time, but the funds increasing, the promoters cast about for still more extensive accommodation, and were so fortunate as to secure a freehold piece of ground—a portion of the present site—in the Fulham Road, and here, in May 1859, Miss Burdett-Coutts, in the presence of a very large company, laid the foundation stone of the first building erected from the designs of Mr. David Mocatta, by Messrs. Lawrence, the cost being somewhere about £7,000. The red Fareham bricks and Ancaster stone which faced the building served rather to give prominence to the institution than to add beauty to what the Brompton boys styled the "red 'us." This building, which contained sixty beds, was soon found inadequate to the requirements, and a large additional piece of ground was purchased, on which the present splendid building now stands, one of the most complete hospitals in London. It contains more than one hundred beds for in-patients, and is fitted with every modern appliance for the comfort of the inmates and the treatment and study of the disease. Mr. Alex. Graham was the architect, and Messrs. E. Lawrence and Co. the builders. The cost, including the additional land, being more than £40,000.

The Present Hospital.

The Interior.

The walls have a hollow interstice of two inches, which helps to ensure dryness and the equalisation of the temperature. The principal wards average about 55 ft. by 25 ft., and are 14 ft. high, the floor-space per bed being no less than 116 ft. They are named The Marsden, Burdett-Coutts, Boyse, Wynn Ellis, etc., etc. The committee-room is a handsome apartment, the walls of which are hung with portraits of deceased committee-men and other supporters of the institution, among the latter being one of Miss—now the Baroness—Burdett-Coutts, taken over twenty years ago; but still, I believe, the portrait of herself which she likes best of any. Then there is another of the founder, each life-size. Passing through the Medical Officers' Rooms, the Pathologist's Room, and the Medical Officers' Committee Room, where ominous consultations are held, the visitor comes to the Burdett-Coutts Ward. There are eight of these large wards and three specials. They are very large, affording over 3,000 cubic feet to each patient, and are airy, exquisitely clean, and orderly. Flowers are everywhere about in plenty, and the wards are about the most cheery and pleasant I was ever in.

Cheerfulness.

By the courtesy of the senior medical officer, Dr. Alex. Marsden, I made a tour of the entire building, and everywhere I found the same attention to order, the same cleanliness, and the same cheerfulness. Cancer is a disease which, perhaps more than any other, tends to weigh down the spirit, and to cast a settled gloom over the unhappy victim. But I must certainly say, judging from what I saw, everything that is possible is done to sustain that mighty Atlas of our burdens—Hope. Some of the wards look out upon the Fulham Road, where an ever-changing scene diverts attention; and at the rear of the hospital is a pretty garden where convalescents can walk without being overlooked by obtrusive eyes.

The Disease.

"The special hospital for which I plead to-day," said the late Archbishop Sumner once, preaching on behalf of this institution, "is dedicated to a malady of a most fearful character, but I would not distress your feelings for one moment by describing the sufferings from the first symptoms of its attack to its close, yet I could wish that the greatness of those sufferings should be more apparent." It is with something of this feeling that I am dealing with this subject. No matter the location of the disease, its painful character makes it about the worst of all bodily ailments. From the establishment of the hospital down to the end of 1884, no less than 19,688 patients had been treated; and of these, 7,007 were dealt with as in-patients, over a half these being discharged either cured or relieved. The disease of course mostly afflicts the female sex. Of the patients treated at the hospital since its foundation, only 4,108 were men, the women numbering 15,580, three-fifths of this latter number suffering from cancer of the breast. Cancer specialists can tell you of extraordinary incidents of female fortitude under operation, of an ideal courage as great as that which Samuel Warren describes in his *Diary of a Physician*, but, before the days of anæsthetics, the removal of cancer was a terrible matter. A lady once, while under operation, said to the surgeon, "Oh, pray don't hurry, but do it thoroughly." Now a breast is removed with ease and cleanness, while the patient is unconscious of a particle of pain.

Cancer Quacks.

Now that the floodgates of knowledge are opening, the days of "cancer curers," *alias* "cancer quacks," are, we hope, gone for ever. No matter how solemn or how awful may be the occasion, the charlatan will press in if the prevailing ignorance of his time will cover his abominable pretences. Not so many years ago cancer quacks often reaped a rich harvest. Their favourite mode of operation was to burn out the cancer, as they called it, but which was much more frequently no cancer at all, by means of caustic. Their fees were as high as their pseudo-remedies were hot. They averaged, as a rule, 50 to 100 guineas when the paste was first put on, another 100 when the "cancer" was "drawn," and another 100 if it ever healed up. During a period lasting from three weeks to six months the victims of these cancer quacks would go through the most frightful agonies, till finally the whole breast would slough off.

Present Work.

A visit to the Cancer Hospital at Brompton will show one how the afflicted value this institution. Patients are drawn hither not only from London and the provinces, but even from Australian colonies, New Zealand, India, etc. The hospital at present contains about 100 beds, while the out-patients average nearly 140 a week. The annual increase in both the in- and out-patients' departments is constant. Last year, for example, the number of new in-patients increased over 22 per cent., and the number of out-patients 33 per cent. upon the numbers of the preceding year. Disease, and *not* the passport of a Governor's letter, is the sole qualification for admission within the walls of this admirable institution.

---

The Eye, Ear, and Throat Hospital, which was opened last August at Halifax, has proved a great boon to the poor, who have not been slow to take advantage of it. During the four months ending December last, 427 patients attended, of whom 190 were discharged cured. So far the little institution has been entirely supported by Dr. Oakley, the honorary surgeon, and two benevolent ladies, but steps are now being taken to organize a Committee of Management, with a view to securing the practical interest of the public.

